# Transient receptor potential V1 modulates neuroinflammation in Parkinson’s disease dementia: Molecular implications for electroacupuncture and rivastigmine

**DOI:** 10.22038/IJBMS.2021.56156.12531

**Published:** 2021-10

**Authors:** Sheng-Ta Tsai, Tzu-Hsuan Wei, Yu-Wan Yang, Ming-Kuei Lu, Shao San, Chon-Haw Tsai, Yi-Wen Lin

**Affiliations:** 1 Department of Neurology, China Medical University Hospital, Taichung, Taiwan; 2 Graduate Institute of Acupuncture Science, College of Chinese Medicine, China Medical University, Taichung, Taiwan; 3 Everflourish Neuroscience and Brain Disease Center, China Medical University Hospital, Taichung, Taiwan; 4 Department of Chinese Medicine, China Medical University Hospital, Taichung, Taiwan; 5 College of Medicine, China Medical University, Taichung, Taiwan; 6 Department of Psychiatry, Taoyuan Psychiatric Center, Ministry of Health and Welfare, Taoyuan, Taiwan; 7 Chinese Medicine Research Center, China Medical University, Taichung, Taiwan

**Keywords:** Electroacupuncture, Hippocampus, Neuroinflammation, Parkinson’s disease – dementia, Rivastigmine, Transient receptor potential- V1

## Abstract

**Objective(s):**

Parkinson’s disease (PD) is a common progressive neurodegeneration disease. Its incidence increases with age and affects about 1% of people over 60. Incidentally, transient receptor potential V1 (TRPV1) and its relation with neuroinflammation in mouse brain has been widely reported.

**Materials and Methods:**

We used 6-hydroxydopamine (6-OHDA) to induce PDD in mice. We then used the Morris water maze and Bio-Plex to test learning and inflammatory mediators in mouse plasma. Western blotting and immunostaining were used to examine TRPV1 pathway in the hippocampus and medial prefrontal cortex (mPFC).

**Results:**

On acquisition days 3 (Control = 4.40 ± 0.8 sec, PDD = 9.82 ± 1.52 sec, EA = 5.04 ± 0.58 sec, Riva = 4.75 ± 0.87 sec; *P*=0.001) and 4, reversal learning days 1, 2, 3 (Control = 2.86 ± 0.46 sec, PDD = 9.80 ± 1.83 sec, EA = 4.6 ± 0.82 sec, Riva = 4.6 ± 1.03 sec;* P*=0.001) and 4, PDD mice showed significantly longer escape latency than the other three groups. Results showed that several cytokines were up-regulated in PDD mice and reversed by EA and rivastigmine. TRPV1 and downstream molecules were up-regulated in PDD mice and further reversed by EA and rivastigmine. Interestingly, α7 nicotinic receptors and parvalbumin levels in both the hippocampus and prefrontal cortex increased in EA-treated mice, but not in rivastigmine-treated mice.

**Conclusion:**

Our results showed that TRPV1 played a role in the modulation of neuroinflammation of PDD, and could potentially be a new target for treatment.

## Introduction

Parkinson’s disease (PD) is the second most common neurodegenerative disease worldwide with increasing rates in elderly populations. Approximately 83% of patients with PD display dementia within 20 years of diagnosis (1). Even in its early stages , 26.7% exhibit mild cognitive impairments (2) which include working memory decline, cognitive inflexibility, and hallucinations (3). These problems impair the patients’ quality of life and impose a significant burden on caregivers (4). Cognitive decline is associated with α-synuclein, tau, and amyloid pathologies and likely involves inflammation and different neurotransmitter systems (5). Because inflammatory responses are amplified by cytokines (IL-1β, TNF-α, IL-6, and IFN-γ) released into the blood via microglial activation (6), neuroinflammation is significantly related to cognitive decline (7).

Accumulating evidence suggests that the transient receptor potential vanilloid type 1 (TRPV1) channel is closely related to immune responses and might be considered a molecular switch for neuroinflammation in many neurodegenerative diseases including PD (8). This protein is a nonselective calcium-permeable cation channel that is highly sensitive to temperature and found in mammals adapted to harsh environments such as polar regions and deserts (9). It is activated by noxious heat, low pH, and animal toxins such as 6-hydroxydopamine (6-OHDA) (10). Brain TRPV1 can potentially detect harmful stimuli and plays a key role in microglia-to-neuron communication. It is highly expressed in microglial cells, which are responsible for inflammation (11) and expressed throughout the central nervous system (CNS), where it potentially supports atypical neurotransmission systems involved in multiple functions through the modulation of neuronal and glial activity (8).

A reduced incidence of PD in smokers has been recognized since the early 1960s (12). Population-based studies show that smokers have an approximately 30%–50% reduced risk of developing PD (13), indicating the importance of nicotinic receptors. The involvement of nicotine receptors could explain the close anatomical relationship between nicotinic cholinergic and dopaminergic neurotransmitter systems in the striatum (14). PD has been considered primarily as a dopaminergic disorder, but multiple CNS systems including cholinergic pathways, are currently thought to be involved in its pathogenesis (15). Several studies using functional imaging, such as proton emission tomography, demonstrate cortical cholinergic dysfunction in patients with PD and cognitive impairment (16). One pathologic investigation has found cholinergic neuronal loss in the nucleus basalis of Meynert in 11 patients with PD, but not in 13 age-matched control subjects (17). Furthermore, clinical trials (18) confirm the treatment efficacy of cholinesterase inhibitors (rivastigmine and donepezil) in patients with PD and cognitive impairment. Such improvement could decrease caregiver distress, including distress resulting from hallucinations (19). Other studies show that α7-nicotinic acetylcholine receptors (α7-nAChRs) have strong links to inflammation and neurodegeneration (20), while others show that α7 nicotinic receptor agonists might decrease neuroinflammations (21).

Acupuncture has been used for at least 3000 years to treat a variety of diseases (22), and complementary and alternative medicine (CAM) with acupuncture in real-world practice is a key component of treating PD worldwide (23). In fact, 63% of patients with PD in Korea (24), 50% in Argentina (25), 39% in Sweden (26), and 25% in Singapore (27) use at least one type of CAM, including acupuncture. More than 20 randomized controlled trials clinically support the efficacy of PD treatment with acupuncture (23). A review of basic studies (28)shows the following mechanisms of acupuncture: neuroprotection, cell proliferation, anti-apoptosis, anti-oxidant, and anti-inflammation. Furthermore a recent study from South Korea demonstrates acupuncture-induced protection of dopaminergic neurons, regulation of gut microbiota, and inhibition of neuroinflammation in mice (29).

In this study, we have shown that neuroinflammatory mediators are up-regulated in PD dementia (PDD). More importantly, the results of our PDD mouse model have shown that TRPV1 and its related molecules play a role in the modulation of neuroinflammation. Because patients prefer either Western medicine or acupuncture, we have compared these treatment types, focusing on cognitive function. We have found that electroacupuncture (EA) and rivastigmine significantly reduced PDD via modulation of TRPV1 signaling. Our data recommend the use of EA and rivastigmine in treating PDD.

## Materials and Methods

***Experimental animals***

We used a newborn subcutaneous 6-OHDA injection mouse model as previously described (30). Thirty-six newborn C57/BL6 mice were randomly assigned to four groups of nine individual animals. The four groups were: control (normal mice), PDD, EA (PDD + electroacupuncture), and Riva (PDD + oral rivastigmine). Mice in the latter three groups were anesthetized with 0.5% isoflurane and given subcutaneous injections of 6-OHDA (100 mg/kg dissolved in 0.1% ascorbic acid in 0.9% NaCl; Sigma, St Louis, Missouri, USA) in the mid-dorsal region for four consecutive days soon after birth. Mice in the control group received vehicle (0.1% ascorbic acid in 0.9% NaCl). Animals were housed in Plexiglas cages with access to standard mouse chow and water ad libitum. Cages were located in a temperature-controlled room (23 °C–27 °C) under a 12:12 hr light-dark cycle (from 6:00 a.m. to 6:00 p.m.) with a relative humidity of 55%–65%. The experiment started at postnatal week eight. Mice weighed 16–23 g at this time. Experimental protocols were approved by the Institute of Animal Care and Use Committee of the China Medical University (Protocol number: CMUIACUC-2020-226), Taiwan, following the Guide for Use of Laboratory Animals (National Academies Press). We tried to minimize the number of animals used and their suffering.

***Electroacupuncture***

Mice in the EA group received electroacupuncture starting on week eight. Animals were treated six times, one time every other day, similar to real-world acupuncture treatment schedules. Stainless steel acupuncture needles (1.5 inch, 32G, Yu Kuang, Taiwan) were inserted bilaterally at KI3 to a depth of 1–2 mm. KI3 was located on the medial aspect of the foot, posterior to the medial malleolus and anterior to the tendon calcaneus (30). Square pulse (100 μs duration) electrical stimulation was delivered for 20 min at 2 Hz and 1 mA. Acupuncture treatments were administered between 11:00 to 14:00.

***Oral rivastigmine***

Mice in group four (Riva) were administered oral rivastigmine starting in week eight, once per day for 12 days. This schedule mimicked everyday use of oral rivastigmine in real-world practice. We used the human liquid formulation of rivastigmine, 120 ml/bottle, containing rivastigmine, 2 mg/ml, produced by Center Laboratories, Inc., Taiwan. We calculated the dose, dissolved the drug in 0.9% NaCl, and administered the solution by gavage.

***Behavioral examination***

**Figure 1:**
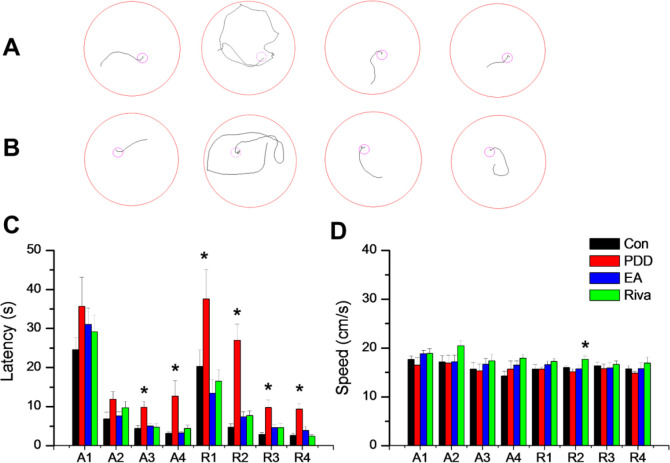
Morris water maze data. (A) Tract recordings of acquisition day 3 (A3), left to right are the four groups of mice: Control (normal mice), PDD (Parkinson’s disease dementia), EA (PDD+ electroacupuncture), and Riva (PDD+ oral rivastigmine). (B) Tract recordings of reversal day 3 (R3), the left to right order is as above. (C) The mean values of escape latency (seconds) and speed (cm/s). The group with asterixis (*) means significantly different from other groups by the one way ANOVA statistics

A circular swimming pool (75 cm in diameter and 22 cm in height) was filled with water, 18 cm deep and maintained at 25 °C. Two principal axes of the maze were defined, with each line bisecting the maze perpendicular to the other to create an imaginary “+.” Ends of each line demarcate the four cardinal directions: North (N), South (S), East (E), and West (W). South (S) was the experimenter’s position, N is the opposite point. We put visual cues around the tank, with white square at the west location, circle in the north location, and triangle in the east location. Locations of visual cues were the same during 16 acquisition and 16 reversal trials for each mouse. A 7 x 7 cm transparent platform was placed 0.5 cm below the surface of the water in the defined area. Data were collected with a digital camera fixed at the top of the room and connected to a computer running Smart V.3 software (TrackMot V.5.45; Signa Technology Company, Taipei, Taiwan). This software measures mouse images to identify the center of its body and track its movement. We first acquired data to test spatial memory of mice. Each day of acquisition included four trials (31). We calculated mean values to generate Figure 1. After four days of acquisition, we changed the transparent platform to the opposite position of the tank to test reversal learning. The reversal learning involved four days, four trials per day. The starting locations of each trial are provided in Table 1. The interval between trials was about five min. Recording started when the camera detected the center of animal mass for two seconds. Recording would stop if the center of mass entered the transparent platform and remained for two seconds. Recording during each trial was 90 sec. If an animal did not reach the platform in time, the experimenter would guide it to the correct position and hold it in place for two seconds. After recording, we used the Smart software to calculate the escape latency, and swimming speed. Daily results are presented in Figure 1 as means and standard errors (SEM). After each trial, we used a heat lamp to warm mice to ensure maintenance of body temperature. The entire behavior test was performed by the same experimenter at the same time (11:00–14:00).

***Bio-Plex ELISA***

After behavior testing, mice were euthanized with 5% isoflurane by inhalation. Blood was collected from the orbital sinus into 3 ml BD Vacutainer glass tubes with 5.4 mg K2 EDTA and 2 ml BD Vacutainer glass tubes with 3 mg sodium fluoride and 6 mg Na2 EDTA. The samples were centrifuged at 1000 rpm/min for 10 min at 25 °C. Separated plasma was collected into 1.5 ml microcentrifuge tubes and stored at −80 °C. Plasma was analyzed using Bio-Plex cytokine assays (BIO-RAD, CA, USA). Four replicates were included.

***Western blot***

After collection of the blood samples, the animals were decapitated, and brains were excised for Western blot analysis. We dissected out bilateral hippocampus and bilateral medial prefrontal cortex (mPFC). The above brain samples were frozen in ice before being stored at -80 °C. Total proteins were prepared by abrasion and lysed in solution of 50 mM Tris-HCl pH 7.4, 250 mM NaCl, 1% NP-40, 5 mM EDTA, 50 mM NaF, 1 mM Na3VO4, 0.02% NaN3, and 1x protease inhibitor cocktail (AMRESCO) before being centrifuged and being added with a bromophenol blue dye. Protein from each sample was loaded on 8% and 12% SDS-Tris glycine gel electrophoresis, followed by transfer onto the PVDF membrane. The membrane was blocked with 5% nonfat milk in TBS-T buffer (10 mM Tris pH7.5, 100 mMNaCl, 0.1% Tween 20) for 1 hr at room temperature; afterward, it was incubated with antibodies (1:1000, Alomone, Jerusalem, Israel): anti-tubulin, anti-GAPDH, anti-TRPV1, anti-pPKA, anti-pPI3K, anti-pPKC, anti-pAkt, anti-pmTOR, anti-pERK, anti-pCREB, anti-α7, and anti-parvalbumin, in TBST with 1% bovine serum albumin. Peroxidase-conjugated anti-mouse, anti-rabbit, or anti-goat antibody (1:5000) was used as a secondary antibody. The protein bands on membranes were visualized by an enhanced chemiluminescencent substrate kit (PIERCE, Rockford, IL, USA) with LAS-3000 Fujifilm (Fuji Photo Film Co. Ltd., Tokyo, Japan). The image densities of the specific bands were quantified using NIH ImageJ software (Bethesda, MD, USA).

***Immunofluorescence***

In each group, we randomly chose three mice to do the immunofluorescence. We euthanized with 5% isoflurane via inhalation, and intracardially perfused with normal saline followed by 4% paraformaldehyde. The brain was immediately dissected and post fixed with 4% paraformaldehyde at 4 °C for 3 days. The tissues were placed in 30% sucrose for cryoprotection overnight at 4 °C. The brain was embedded in optimal cutting temperature (OCT) compound and rapidly frozen using liquid nitrogen before storing the tissues at -80 °C. Frozen segments were cut at 20-um width on a cryostat then instantaneously placed on glass slides. The samples were fixed with 4% paraformaldehyde, and then incubated with blocking solution, consisting of 3% BSA, 0.1% Triton X-100, and 0.02% sodium azide, for 1 hr at room temperature. After blocking, the samples were incubated with primary antibody (1:200, Alomone, Jerusalem, Israel), TRPV1, prepared in 1% bovine serum albumin solution at 4 °C overnight. Afterward, the samples were incubated with the secondary antibody (1:500), 488-conjugated AffiniPure donkey anti-rabbit IgG (H +L), 594-conjugated AffiniPure donkey anti-goat IgG (H + L), and Peroxidase-conjugated AffiniPure donkey anti-mouse IgG (H + L) for 2 hr at room temperature before being fixed with cover slips for immunofluorescence visualization. The samples were observed by an epi-fluorescent microscope (Olympus, BX-51, Tokyo, Japan) with 20x numerical aperture (NA=0.4) objective. The images were analyzed by NIH ImageJ software (Bethesda, MD, USA).

***Data analysis***

The data of this study have been expressed as the mean ± standard errors (SEM). We used the one-way ANOVA, then post hoc Tukey’s test to calculate *P*-values for continuous variables. All statistical analyses were performed using Origin (OriginLab Corporation, Northampton, Massachusetts, USA), version 8. The threshold for statistical significance was set at *P*=0.05 based on a two-sided test.

## Results

Electroacupuncture (EA) and rivastigmine significantly reversed 6-OHDA induced spatial and reversal learning dysfunction, but not motor function in a PDD mouse model.

We used a Morris water maze for behavioral tests. In the first four days, acquisition behavior training was performed. Escape latency in each group of mice decreased day-to-day (Figure 1). On acquisition days 3 (Control = 4.40 ± 0.8 sec, PDD = 9.82 ± 1.52 sec, EA = 5.04 ± 0.58 sec, Riva = 4.75 ± 0.87 sec; *P*=0.001) and 4, PDD mice showed significantly longer escape latency than other treated mice, indicating that PDD mice displayed impaired spatial memory. This impairment was reversed by EA and oral rivastigmine. After acquisition, we put the hidden platform in an opposite area to test reversal learning. PDD mice showed prolonged escape latency over all four reversal days (R1-R4). On reversal day 3, the escape latencies were: Control = 2.86 ± 0.46 sec, PDD = 9.80 ± 1.83 sec, EA = 4.6 ± 0.82 sec, Riva = 4.6 ± 1.03 sec; *P*=0.001. Similarly, learning impairment was reversed by EA or oral rivastigmine (Figure 1C). All mice exhibited similar swimming speed on seven of eight days. Only on reversal day 2 (R2), mice in the drug group showed faster swimming (Figure 1D). PDD mice apparently did not suffer motor dysfunction as expressed by bradykinesia. Thus, prolonged escape latency was solely due to cognitive decline.

Inflammatory cytokines were increased in PDD mice plasma and further attenuated through EA or rivastigmine treatment. 

**Figure 2:**
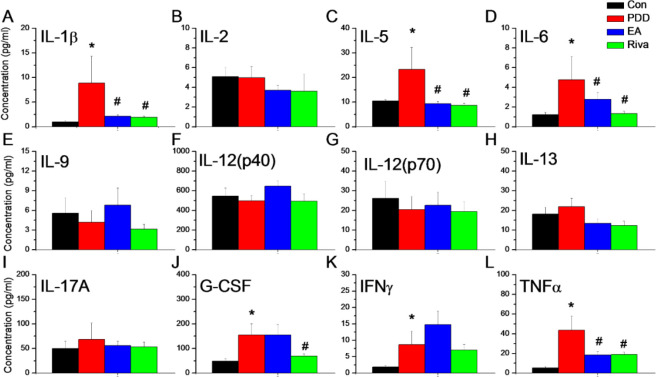
The expression of inflammatory cytokines in mice plasma. (A) IL-1β, (B) IL-2, (C) IL-5, (D) IL-6, (E) IL-9, (F) IL-12 (p40), (G) IL-12 (p70), (H) IL-13, (I) IL-17α, (J) G-CSF, (K) IFN-γ, (L) TNF-α.*means significant difference with the control group. #means significant difference with the PDD group

IL: Interleukin; G-CSF: Granulocyte colony-stimulating factor; IFN: Interferon; TNF: Tumor necrosis factor

We next used the Bio-Plex ELISA to examine pro- and anti-inflammatory cytokines in mice plasma (IL-1β, IL-2, IL-4, IL-5, IL-6, IL-9, IL-10, IL-12 (p40), IL-12 (p70), IL-13, IL-17α, G-CSF, IFN-α, TNF-α, MCP-1, MIP-1α, MIP-1β, RANTES, Eotaxin, GM-CSF, and KC.). Several cytokines, IL-1β, IL-5, IL-6, G-CSF, IFN-γ, and TNF-α were up-regulated in PDD mice; EA significantly attenuated IL-1β, IL-5, IL-6, and TNF-α expression in mouse plasma. Further, rivastigmine reliably reduced the up-regulation of IL-1β, IL-5, IL-6, G-CSF, and TNF. Data are presented in Figure 2.

The effect of EA and rivastigmine treatment on TRPV1 and downstream signaling in the hippocampus and PFC.

**Figure 3:**
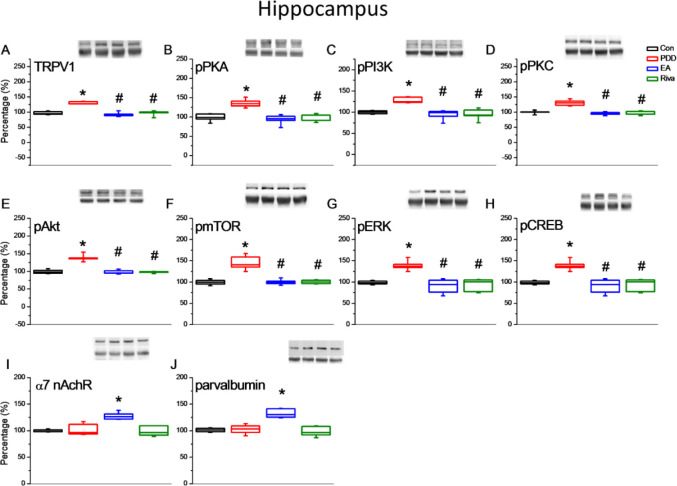
Expression levels of TRPV1-associated signaling pathways in the mice hippocampus. (A) TRPV1, (B) pPKA, (C) pPI3K, (D) pPKC, (E) pAkt, (F) pmTOR, (G) pERK, (H) pCREB, (I) α7 nicotinic receptor, and (J) Parvalbumin expression levels in Con, PDD, EA, Riva. Con: normal mice; PDD: Parkinson’s disease dementia mice; EA: PDD+ EA. Riva: PDD + oral rivastigmine. Each group n= 6

**Figure 4:**
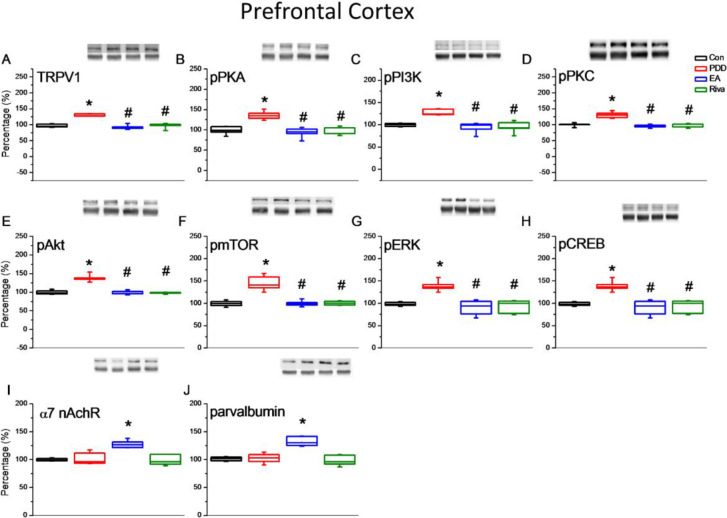
Expression levels of TRPV1-associated signaling pathways in the mice prefrontal cortex. (A) TRPV1, (B) pPKA, (C) pPI3K, (D) pPKC, (E) pAkt, (F) pmTOR, (G) pERK, (H) pCREB, (I) α7 nicotinic receptor, and (J) Parvalbumin expression levels in Con, PDD, EA, Riva. Con: normal mice; PDD: Parkinson’s disease dementia mice; EA: PDD + EA. Riva: PDD + oral rivastigmine. Each group n= 6

Behavior tests showed impairment in both spatial learning and cognitive flexibility in PDD mice. Associated changes in proteins in brain samples were assessed by Western blotting. We focused on the hippocampus for spatial learning and the PFC for reversal learning. In both areas, TRPV1 and downstream molecules (pPKA, pPI3K, pPKC, pAkt, pmTOR, pERK, and pCREB) were up-regulated in PDD group mice. This increase in expression was reversed by EA and oral rivastigmine. Interestingly, EA treated mice showed an increase in α7 nicotinic receptors and parvalbumin level in these brain areas (Figures 3 and 4).

The effect of EA or rivastigmine treatment on TRPV1 expression in the hippocampus and PFC via immunofluorescence technique.

**Figure 5:**
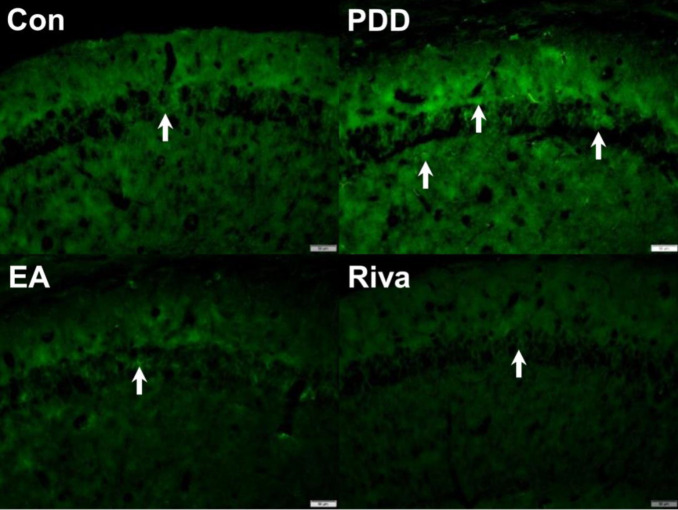
Immunofluorescence staining of TRPV1 protein in the hippocampal CA1 area. Con: Control, PDD: Parkinson’s disease dementia, EA: PDD + EA, Riva: PDD + rivastigmine. Each group n= 3. Scale bar in the right lower corner of each picture represents 50 µm. White arrows indicate TRPV1-positive neurons

**Figure 6:**
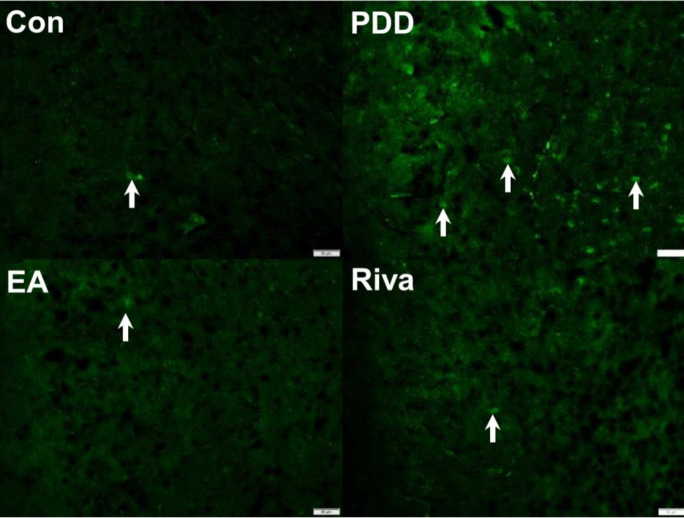
Immunofluorescence staining of TRPV1 protein expression in the prefrontal cortex. Con: Control, PDD: Parkinson’s disease dementia, EA: PDD + EA, Riva: PDD + rivastigmine. Each group n= 3. Scale bar (in the right lower part of each picture) is 50 µm. The white arrows indicate TRPV1-positive neurons

Western blotting analysis showed TRPV1 up-regulation in the hippocampus and PFC. We used immunofluorescence to stain TRPV1 positive cells in the hippocampus (Figure 5) and PFC (Figure 6). We observed consistent Western blotting results that showed an increase in TRPV1 expression in PDD mice. This increase could be reversed by EA or oral rivastigmine.

**Table 1:** Morris water maze spatial (hidden platform) start positions. "A mouse had four trials per day to swim toward the hidden platform, starting from different locations. This method reduced the data variation of a single trial

Acquisition: hidden platform at SW				
Day	Trial 1	Trial 2	Trial 3	Trial 4
1 (A1)	N	E	SE	NW
2 (A2)	SE	N	NW	E
3 (A3)	NW	SE	E	N
4 (A4)	E	NW	N	SE
Reversal: hidden platform at NE				
Day	Trial 1	Trial 2	Trial 3	Trial 4
1 (R1)	S	W	NW	SE
2 (R2)	NW	S	SE	W
3 (R3)	SE	NW	W	S
4 (R4)	W	SE	S	NW

A: acquisition; R: reversal; N: North; E: East; S: South; W: West, SW: Southwest; SE: Southeast; NW: Northwest; NE: Northeast

*P<0.05 compared with the normal group. #*P*<0.05 compared with the PDD group. The Western blot bands at the top show the target protein. The lower bands are internal controls (GAPDH in α 7 nicotinic receptor, and α-tubulin in others)

*P<0.05 compared with the normal group. #*P*<0.05 compared with the PDD group. The Western blot bands at the top show the target protein. The lower bands are internal controls (GAPDH in α7 nicotinic receptor, and α-tubulin in others)

**Figure 7:**
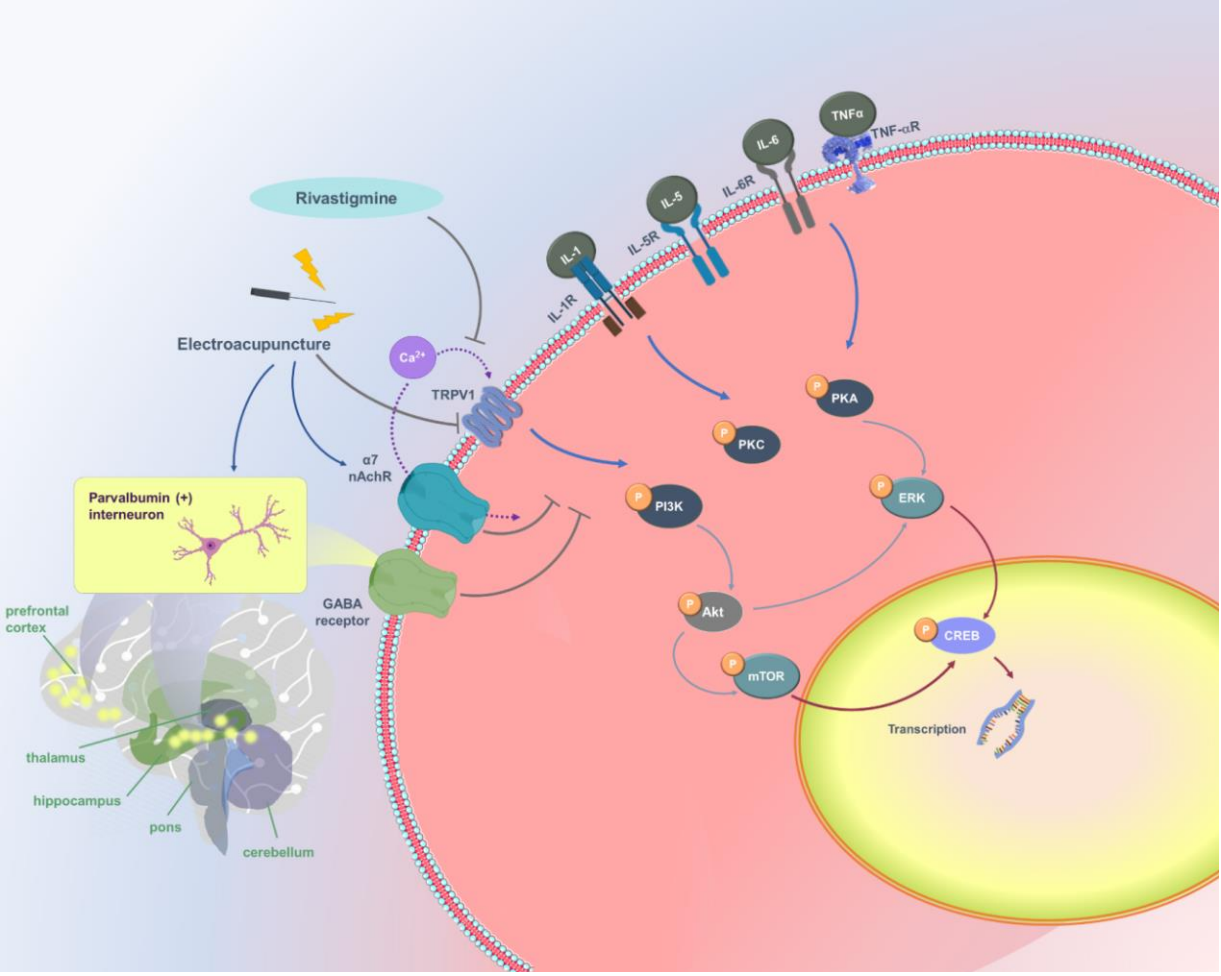
TRPV1 and related molecular pathways

**Table 2:** Acupuncture and TRPV1

Disease	Animal	Disease model	Target region	Acupoint	Acupuncture functions	First author	Year
No disease	Rat	Normal rat	Acupoint: subepidermal nerve fibers	BL40	Increase TRPV1	Therese S. Abraham	2011 (44)
No disease	Mice	Normal mice and TRPV1 knockout mice	DRG, spinal cord, somatosensory cortex	ST36	Increase TRPV1	Hsiao-Chun Chen	2018 (45)
Obesity	Mice	Normal mice (EA mice had less weight gain)	DRG, spinal cord	ST36	Increase TRPV1	Monchanok Choowanthanapakorn	2015 (46)
Chronic pain and depression	Mice	Intermittent cold-stress	mPFC, hippocampus and PAG	ST36	Increase TRPV1	Yi-Wen Lin	2020 (47)
Inflammatory pain	Mice	CFA intraplantar injection in the right hind paw	Muscle and epimysium at ST36 area	ST36	Increase TRPV1	Shu-Yih Wu	2014 (48)
		acidic saline (pH 4.0) injection into the right gastrocnemius muscle (GM)	DRG, spinal cord	ST36	Decrease TRPV1	Jaung-Geng Lin	2015 (49)
			DRG, spinal cord, thalamus, somatosensory cortex	ST36	Decrease TRPV1	Chia-Ming Yen	2020 (50)
		CFA intraplantar injection in the right hind paw	DRG	ST36	Decrease TRPV1	Wei-Hsin Chen	2012 (51)
			DRG	ST36, ST37	Decrease TRPV1	Kung-Wen Lu	2016 (52)
			DRG, spinal cord	ST36	Decrease TRPV1	Jun Yang	2017 (53)
			DRG, spinal cord	ST36	Decrease TRPV1	Hsien-Yin Liao	2017 (54)
			PFC, hypothalamus, PAG	LI4	Decrease TRPV1	Chia-Ming Yen	2019 (55)
			Thalamus, amygdala and somatosensory cortex	ST36	Decrease TRPV1	Hsin-Cheng Hsu	2020 (56)
			Cerebellum	ST36	Decrease TRPV1	Chanya Inprasit	2020 (57)
Motion sickness	Mice	Rotation at a velocity of 80 rpm continuously for 40 mins, one time per day, total four days	Thalamus and hypothalamus	PC6	Decrease TRPV1	Chanya Inprasit	2018 (58)
Sympathoexcitatory cardiovascular reflex	Rat	Gastric distention induced blood pressure increase	DRG	PC5, PC6	Decrease TRPV1	Zhi-Ling Guo	2018 (59)
Inflammatory bowel syndrome	Mice	Transanal Zymosan injection to induce colorectal distension	Colorectum	ST36, ST37	Decrease TRPV1	Shao-Jun Wang	2012 (60)
Parkinson's disease dementia	Mice	6-OHDA subcutaneous injection after birth	Hippocampus and PFC	KI3	Decrease TRPV1	Sheng-Ta Tsai	this paper

TRPV1: Transient receptor potential V1; DRG: Dorsal Root Ganglia; EA: Electroacupuncture; CFA: Complete Freund's Adjuvant; PFC: Prefrontal Cortex; PAG: Periaqueductal Gray

## Discussion

A 2019 review article summarized recent studies of neuroinflammation in PD-associated neurodegeneration. Proinflammatory cytokines(IL-1β, IL-6, and TNF-α), mediated by the microglia and astrocytes play an important role in this process (32). Using 6-OHDA to induce neuroinflammation in a PDD mouse model, we increased plasma proinflammatory cytokine concentrations of IL-1β , IL-5, IL-6, and TNF-α. Consistent with a previous study, neuroinflammation paralleled TRPV1 activation in the hippocampus and PFC (8). The summary of our finding is shown in Figure 7.

Interestingly, out of the 18 studies we reviewed that focused on the modulation of TRPV1 via acupuncture (Table 2), 13 studies showed that acupuncture decreased TRPV1 expression to relieve symptoms, while the other five reported an increase in TRPV1 expression. This discrepancy may be due to the bidirectional modulations of both acupuncture (33) and neuroinflammation byTRPV1 (8). Since most animal studies on PD investigated motor symptoms such as bradykinesia and rigidity, we focused on two cognitive domains: spatial memory and cognitive flexibility. Previous research used the same animal PDD model (30) and showed that electroacupuncture rescued learning and long-term potentiation deficits. Authors reported that electroacupuncture (EA) on the bilateral KI3 reduced neuronal excitotoxicity by regulating N-methyl-d-aspartate (NMDA) receptor functions. We used a similar method and analyzed TRPV1 and related signaling, along with a behavior test for reversal learning to investigate cognitive inflexibility in PDD mice.

Some patients with PD clinically display rigid thinking and have difficulty altering their ideas. Cools *et al*. focused on cognitive flexibility (34) and used a strict method to simplify concept formation, learning, working memory, and a general slowing of cognitive processes. They reported strong evidence of cognitive inflexibility in patients with PD, with disrupted interactions between the frontal cortex and striatum. Another recent study showed that the dysfunction of parvalbumin (PV)-positive GABAergic interneurons (PVIs) within the PFC was associated with cognitive inflexibility (35). Parvalbumin is a calcium-binding low molecular weight protein, typically 9–11 kDa (36). We examined parvalbumin in both the PFC and hippocampus. Interestingly, we found that parvalbumin levels increased in mice treated with electroacupuncture but not after oral administration of rivastigmine. This finding was consistent with reports that electroacupuncture alleviates anxiety-like behavior in adult mice (37). Another study investigated the disrupted balance between inhibition, such as parvalbumin-positive GABAergic interneurons, and excitation within the neuronal networks for acupuncture and epilepsy (38). That study showed that parvalbumin was more GABAergic, while TRPV1 activation was more glutamatergic (39). Because of this, we speculated that increased GABAergic effects of electroacupuncture reduced glutamatergic effects of TRPV1 and thus improved cognitive flexibility of mice.

Another difference between EA and oral rivastigmine is the effect on swimming speed. We found that mice administered with rivastigmine swam faster on all eight testing days. However, only results from reversal day 2 (R2) showed statistical significance (*P*=0.03). We encountered similar results in phase 2 clinical studies of patients with PD treated with rivastigmine (40). This treatment improved gait stability and might reduce fall frequency. Other studies (41) found that patients with PD need to concentrate to compensate for impaired gait stability and that oral rivastigmine might improve gait by improving cognitive function and attention (42).

Clinical studies showed that LR3 (Tai Chong) is the most common acupoint for PD treatment, other than GB34, GV20, EX-HN1, GB20, LI11, ST36, and KI3 (Tai Xi)(23). Using functional MRI to evaluate acupuncture effects in the brain, KI3 was shown to improve cognitive function in patients in human studies (43). Similarly, bilateral EA using KI3 showed positive effects in the hippocampus in a previous PDD mouse study (30). According to traditional Chinese medical history, although the pathological location of cognitive decline is in the brain, an essential factor lies in the kidney, hence, KI3 (Tai xi) is considered as a primary acupoint used clinically for treating cognitive disorders.

## Conclusion

Our study has found that PDD involves neuroinflammation and that the modulation of TRPV1 and related signaling via treatment with EA and oral rivastigmine might alleviate this inflammation. Therefore, TRPV1 may be a target for the treatment of patients with PDD. Since the treatments used here affect different molecular pathways, further studies are needed to clarify their difference in detail. 

## Data Availability

The data used to support the findings of this study are available from the corresponding author upon request.

## Authors' Contributions

STT and THW Conceptualization, methodology; YWY, MKL, and Shao San Software, Data curation, writing the original draft, visualization, and investigation. CHT and YWL Supervision, validation, writing, review and editing.

## Conflicts of Interest

The authors declare no conflicts of interest.
